# HNF1B Mutations Are Associated With a Gitelman-like Tubulopathy That Develops During Childhood

**DOI:** 10.1016/j.ekir.2019.05.019

**Published:** 2019-05-29

**Authors:** Shazia Adalat, Wesley N. Hayes, William A. Bryant, John Booth, Adrian S. Woolf, Robert Kleta, Sandra Subtil, Rhian Clissold, Kevin Colclough, Sian Ellard, Detlef Bockenhauer

**Affiliations:** 1 Evelina Children’s Hospital, London, United Kingdom; 2 UCL Department of Renal Medicine, London, United Kingdom; 3 Great Ormond Street Hospital for Children NHS Foundation Trust, London, United Kingdom; 4 Division of Cell Matrix Biology and Regenerative Medicine, School of Biological Sciences, Faculty of Biology Medicine and Health, University of Manchester, United Kingdom; 5 Royal Manchester Children’s Hospital, Manchester University NHS Foundation Trust, Manchester Academic Health Science Centre, Manchester, United Kingdom; 6 St Mary’s Hospital, London, United Kingdom; 7 Royal Devon and Exeter NHS Foundation Trust, Exeter, United Kingdom; 8 Department of Molecular Genetics, Royal Devon & Exeter NHS Foundation Trust, Exeter, United Kingdom; 9 Institute of Biomedical and Clinical Science, College of Medicine and Health, University of Exeter, Exeter, United Kingdom

**Keywords:** alkalosis, children, HNF1B, hypokalemia, hypomagnesemia, renal tubular function

## Abstract

**Background:**

Mutations in the transcription factor hepatocyte nuclear factor 1B (HNF1B) are the most common inherited cause of renal malformations, yet also associated with renal tubular dysfunction, most prominently magnesium wasting with hypomagnesemia. The presence of hypomagnesemia has been proposed to help select appropriate patients for genetic testing. Yet, in a large cohort, hypomagnesemia was discriminatory only in adult, but not in pediatric patients. We therefore investigated whether hypomagnesemia and other biochemical changes develop with age.

**Methods:**

We performed a retrospective analysis of clinical, biochemical, and genetic results of pediatric patients with renal malformations tested for *HNF1B* mutations, separated into 4 age groups. Values were excluded if concurrent estimated glomerular filtration rate (eGFR) was <30 ml/min per 1.73 m^2^, or after transplantation.

**Results:**

A total of 199 patients underwent *HNF1B* genetic testing and mutations were identified in 52 (mut+). The eGFRs were comparable between mut+ and mut− in any age group. Although median plasma magnesium concentrations differed significantly between mut+ and mut− patients in all age groups, overt hypomagnesemia was not present until the second half of childhood in the mut+ group. There was also a significant difference in median potassium concentrations in late childhood with lower values in the mut+ cohort.

**Conclusions:**

The abnormal tubular electrolyte handling associated with *HNF1B* mutations develops with age and is not restricted to magnesium, but consistent with a more generalized dysfunction of the distal convoluted tubule, reminiscent of Gitelman syndrome. The absence of these abnormalities in early childhood should not preclude *HNF1B* mutations from diagnostic considerations.

HNF1B is a transcription factor highly expressed in the developing kidney, genital tract, pancreas, and liver.^1^ Heterozygote mutations in the encoding gene lead to autosomal dominant tubulointerstitial kidney disease–HNF1B,^2^ and besides cystic kidney disease the clinical spectrum can include renal malformations, diabetes, genital tract abnormalities, exocrine pancreatic insufficency, and gout.^3^, ^4^ The high clinical variability, a spontaneous mutation rate of approximately 50%, and variable penetrance hamper clinical diagnosis.^5^

Previously, we reported hypomagnesemia as part of the clinical spectrum, suggesting a role for HNF1B not only in morphological renal development, but also in the maintenance of tubular function.^6^ To rationalize patient selection for genetic testing, a clinical tool had subsequently been proposed that predicts the presence of *HNF1B* mutations based on a score derived from several clinical features, including hypomagnesemia.^7^ Yet, when this score was applied to a large cohort in the United Kingdom, which included patients investigated here, hypomagnesemia was found to be discriminatory only in adult patients, not in children.^8^ In another predominantly pediatric cohort, hypomagnesemia was present in only a quarter of patients with HNF1B mutations.^9^

HNF1B-associated hypomagnesemia is associated with altered transactivation of the gamma-subunit of the Na^+^-K^+^-ATPase in the distal convoluted tubule (DCT), which regulates epithelial ion transport.^6^, ^10^ Impaired general transport activity in the DCT is usually associated with a Gitelman-like tubulopathy, consisting of hypokalemic hypochloraemic alkalosis with hypocalciuria, in addition to hypomagnesemia.^11^ We prevously reported hypocalciuria in children with *HNF1B* mutations, but had not investigated hypokalemia, hypochloremia, or alkalosis, although hypokalemia has previously been reported in adult patients.^6^, ^12^

We hypothesized that the electrolyte abnormalities associated with *HNF1B* mutations develop during childhood and therefore the application of the score in younger children may wrongly predict the absence of a mutation. We thus decided to assess this in our cohort of children with renal malformations with and without identified *HNF1B* mutations.

## Methods

### Patients

**Figure 1:**
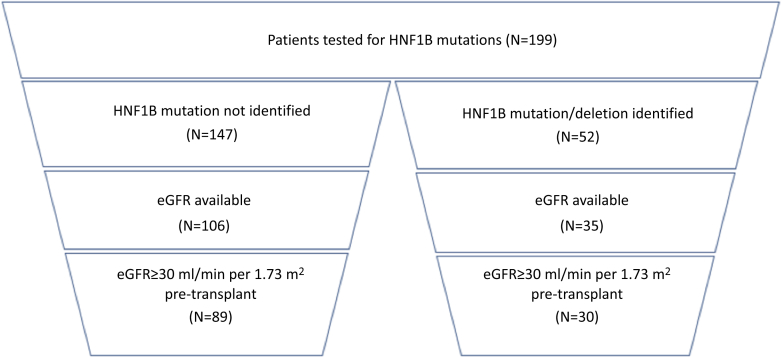
Funnel diagram of patient identification. Shown is the number (*n*) of patients included in the analysis. A total of 199 patients with renal malformations were identified that had testing for *HNF1B* performed. After exclusion of those without an available glomerular filtration rate (GFR; measured or estimated) and those with a GFR of <30 ml/min per 1.73 m^2^, 30 mut+ and 89 mut− patients remained with biochemical values suitable for analysis. eGFR, estimated glomerular filtration rate.

We performed a retrospective analysis of clinical, biochemical, and genetic results of patients tested for *HNF1B* mutations seen at Great Ormond Street Hospital with chronic kidney disease stage 1 to 3 between 2000 and 2017. Mutation analysis had been performed at the discretion of the individual treating physician and patients’ leucocyte DNA was screened for *HNF1B* mutations as described previously.^5^, ^6^, ^13^, ^14^ An overview of patient and data selection is given in Figure 1.

### Biochemical Data

Biochemical and clinical data were retrieved from relevant hospital databases. Results were anonymized and analyzed.

Plasma and urine biochemistries were obtained and compared between those with confirmed *HNF1B* mutations (mut+) and those without (mut−).

Formal measured glomerular filtration rates were used if available. Clinical parameters were otherwise used to calculate eGFR using the Schwartz-Haycock formula with the factor k specifically adapted to our hospital laboratory, as described previously.^15^ Results with a concurrent measured or eGFR below 30 ml/min per 1.73 m^2^ and all results post transplant were excluded.

All available results for the following biochemical parameters were obtained: plasma concentrations of sodium, potassium, magnesium, chloride, calcium, phosphate, and bicarbonate (measured as total CO_2_), as well as urine calcium/creatinine ratios. As the normal range for urine calcium/creatinine ratio changes with age, ratios were normalized to the respective upper limit of the normal range, as described previously.^16^

Results were then separated into 4 age groups: 0 to <4.5, 4.5 to <9, 9 to <13.5, and 13.5 to 18 years of age.

If within 1 age group more than 1 value per biochemical parameter was availble for an individual patient, we calculated median values to exclude bias from overrepresentation of patients with more available results.

### HNF1B Score

We identified those patients who had been included in a previous study of the HNF1B score^8^ and who were included in this study because of available biochemistries. The previously calculated HNF1B score was retrieved and adjusted using the latest plasma magnesium concentration.

### Statistical Analysis

Statistical analysis was performed using R (Vienna, Austria).^17^ Nonparametric Wilcoxon and Fisher’s exact tests were implemented for statistical analysis.

We used Fisher’s exact test (2-tailed) to assess the siginificance of the difference in number of patients with abnormal results, that is, below (Mg, K, and Cl) or above (bicarbonate) the respective reference range between the mut+ and mut− groups.

We used the Wilcoxon test to compare all the numerical values in the respective groups to determine the signficance of the difference in the medians.

## Results

### Patients

A total of 199 children had genetic testing for HNF1B performed, 72 of whom had also been included in our initial report of the association of hypomagnesemia with HNF1B.^6^ In 52 patients (26%), mutations were identified, constituting the mut+ cohort, most (*n* = 33) being whole gene deletions. As in our previous review, no difference in electrolyte patterns could be seen between patients with intragenic mutations versus whole gene deletions (data not shown).^6^

The remaining 147 patients constituted the mut− cohort. There was no significant (*P* = 0.3–0.7) difference between the 2 cohorts (mut+ vs. mut−) with respect to glomerular filtration rates in any age group (Table 1).

**Table 1:** Plasma and urine biochemistry values

Parameters by age, yr	*n* (mut+)	mut+ Median (range)	mut− Median (range)	Wilcoxon
**GFR**				
0–4.5	76 (20)	61 (43–91)	70 (32–117)	0.4
4.5–9.0	51 (11)	69 (31–95)	60 (30–115)	0.4
9.0–13.5	43 (11)	71 (43–101)	62 (30–106)	0.3
13.5–18.0	26 (5)	59 (36–78)	62 (32–109)	0.7
**Magnesium**	Normal >0.66 (<9 yr) or 0.7 (>9 yr) mmol/l			
0–4.5	71 (18)	0.76 (0.53–0.88)	0.83 (0.61–1.11)	0.004^a^
4.5–9.0	49 (10)	0.69 (0.52–0.77)	0.77 (0.52–0.97)	0.005
9.0–13.5	40 (10)	0.57 (0.45–0.77)	0.81 (0.64–0.96)	0.00002^a^
13.5–18.0	24 (5)	0.53 (0.5–0.64)	0.84 (0.61–1.01)	0.001^a^
**Potassium**	Normal >3.5 mmol/l			
0–4.5	73 (20)	4.2 (3.8–5.3)	4.2 (3.8–5.3)	0.4
4.5–9.0	51 (11)	4.1 (3.7–4.6)	4.1 (2.8–5.6)	0.09
9.0–13.5	43 (11)	3.9 (3.3–5.1)	4.2 (3.3–5.0)	0.09
13.5–18.0	26 (5)	3.6 (3.4–4.0)	4.2 (3.4–4.9)	0.02^a^
**Chloride**	Normal >100 mmol/l			
0–4.5	14 (3)	106 (101–106)	105 (94–108)	1
4.5–9.0	14 (4)	104 (101–107)	106 (101–112)	0.9
9.0–13.5	16 (4)	101 (101–109)	104 (97–109)	0.26
13.5–18.0	6 (3)	100 (99–100)	103 (101–109)	0.14
**Bicarbonate**	Normal <30 mmol/l			
0–4.5	71 (20)	24 (16–27)	22 (15–34)	0.11
4.5–9.0	48 (11)	25 (22–29)	25 (18–29)	0.09
9.0–13.5	41 (11)	28 (24–31)	23 (18–30)	0.0002^a^
13.5–18.0	23 (5)	27 (25–31)	24 (18–28)	0.0007^a^
**Normalized UCCR**	Normal <1			
0–4.5	7 (2)	0.07 (0.07–0.07)	0.5 (0.3–1.1)	0.3
4.5–9.0	5 (3)	0.1 (0.07–0.1)	0.9 (0.1–1.8)	0.7
9.0–13.5	1 (0)	No results	0.5	n/a
13.5–18.0	9 (3)	0.07(0.04–0.2)	0.7 (0.2–1.1)	0.07
All ages	22 (8)	0.07 (0.04–0.2)	0.5 (0.1–1.8)	0.0005^a^

GFR, glomerular filtration rate; n/a, not applicable; UCCR, urine calcium/creatinine ratio.

Shown are pertinent plasma and urine biochemistries separated according to *HNF1B* mutations status (mut+ vs. mut−) and according to the 4 age groups. The respective lower or upper limit of normal is indicated for each electrolyte concentration. The number of patients with available data “*n*” according to mutation status is provided in the second column, with the number of mut+ patients in parentheses. Note that patients can be represented in more than 1 age group, if data were available.

The Wilcoxon test compares the median values between the mut+ and mut− groups. The UCCR is normalized to the respective upper limit of normal for age to allow comparison across the age groups. There were too few measurements for this parameter to allow robust statistical comparison in the individual age groups.

Note that median magnesium values are significantly different between mut+ and mut− patients in all age groups. Median bicarbonate and potassium values were also significantly different but only in late childhood.

a Significant (*P* < 0.05) values.

### Age

Both cohorts were comparable with respect to median age at first (2.19 years, range 0.15–15.9 [mut+] vs. 2.8 years, range 0.02–17.1 [mut−]) and last available blood test (8.9 years, range 0.21–17.3 [mut+] vs. 7.3 years, range 1.1–17.4 [mut− ]).

### Magnesium

**Figure 2:**
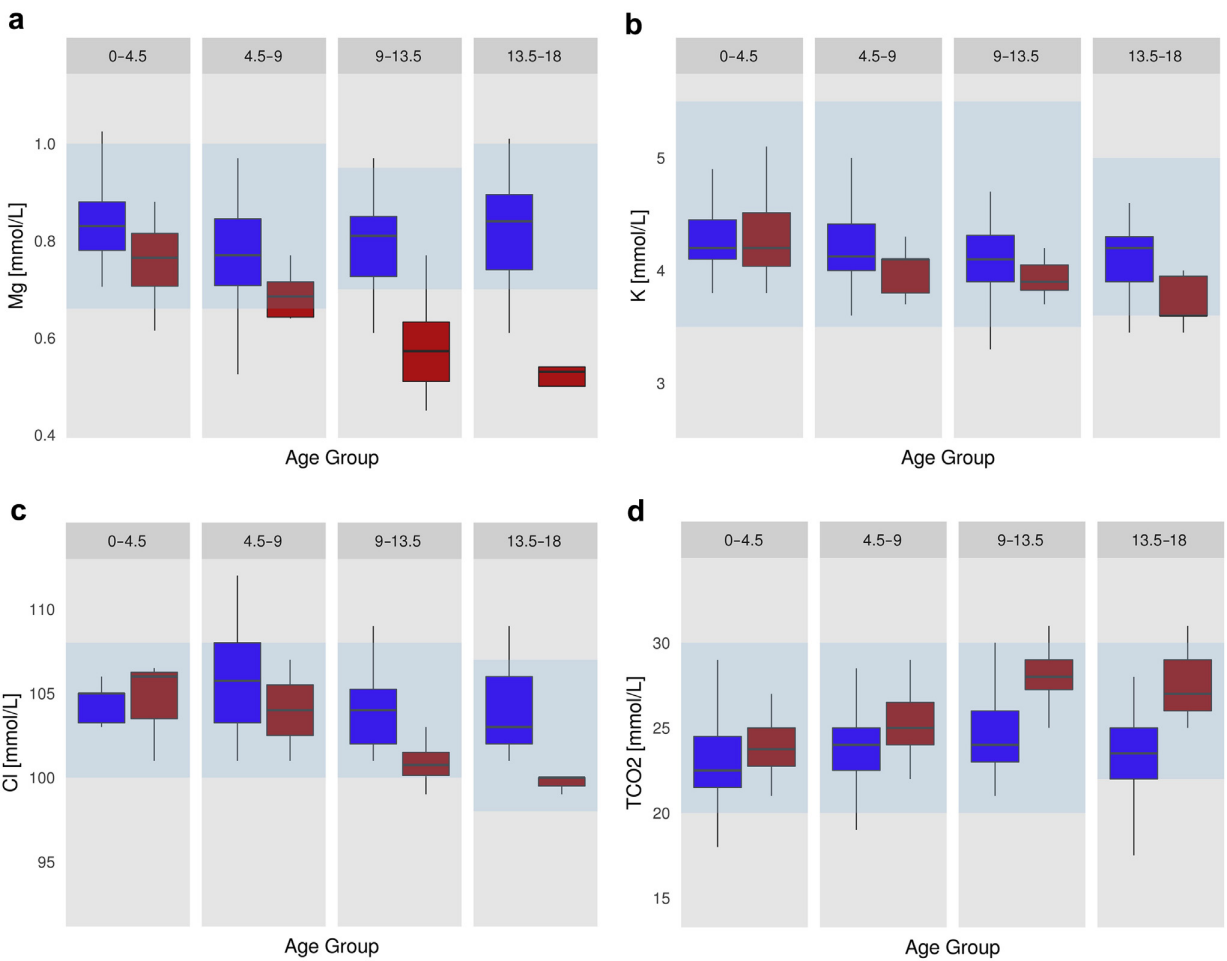
Plasma electrolyte abnormalities in mut+ patients develop over time. Shown are box plots for the plasma concentrations of (a) magnesium (Mg), (b) potassium (K), (c) chloride (Cl), and (d) bicarbonate (TCO2) according to the 4 age groups. Box plot graphs represent the median and interquartile range (IQR); the upper and lower whiskers include data points within 1.5 × IQR. Outliers are plotted individually. The blue boxes represent the mut− group, and the red boxes represent mut+. The respective normal range is represented by the transparent blue boxes. Note the development with increasing age of a Gitelman-like tubulopathy with hypomagnesemia and hypokalemic, hypochloremic metabolic alkalosis in the mut+ group. For number of patients for each plot and results of statistical comparisons, see Table 1.

**Figure 3:**
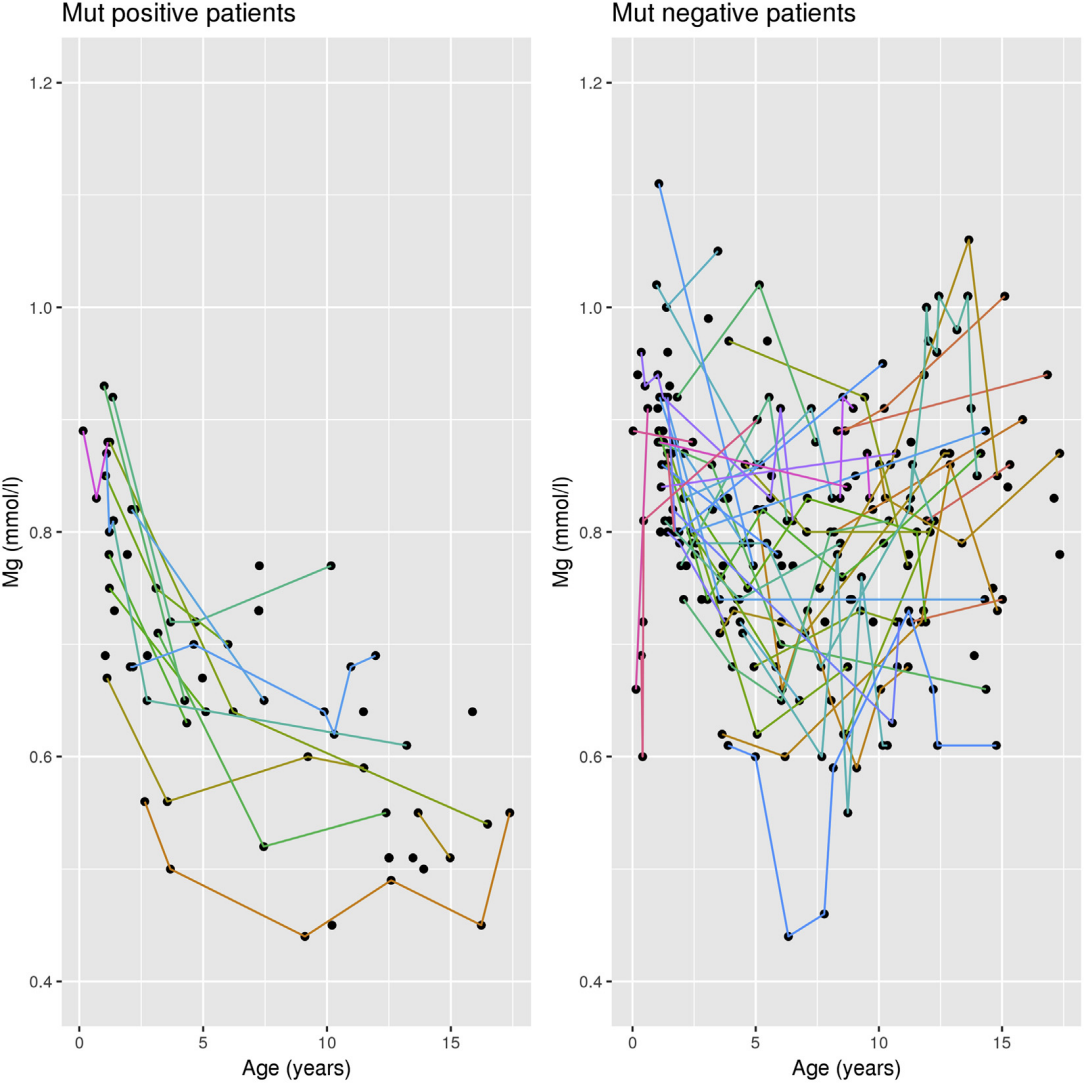
Magnesium levels in individual patients over time. Plotted are all plasma magnesium measurements included in the analysis with individual patients represented by colored lines, if more than 1 measurement was available. Note the decreasing plasma magnesium levels in the mut+ group, whereas no such trend is noticeable in the mut− group.

Table 1 summarizes the analysis of median biochemical concentrations assessed by mutation status and age group and they are graphically represented in Figure 2. Individual plasma magnesium measurements are plotted in Figure 3. Of note, although median plasma magnesium values differed significantly between mut+ and mut− in every age group, the number of patients with overt hypomagnesemia (median magnesium concentration below 0.7 mmol/l) became significantly different between the mutation groups only in the second half of childhood (Table 2). The median age at which hypomagnesemia was first noted in mut+ patients was 10.0 years (1.05–17.4 years).

**Table 2:** Comparison of hypomagnesemia in HNF1B mut+ and mut− groups

Age (yr)	*HNF1B* mutation	Hypomagnesemia patients, *n* (%)	Normomagnesemia patients, *n* (%)	Fisher’s exact comparison
0–4.5	+	4 (22)	14 (78)	*P* = 0.26
	−	2 (4)	51 (96)	
4.5–9.0	+	5 (50)	5 (50)	*P* = 0.18
	−	9 (23)	30 (77)	
9.0–13.5	+	9 (90)	1 (10)	*P* = 0.0001^a^
	−	5 (17)	25 (83)	
13.5–18.0	+	5 (100)	0 (0)	*P* = 0.02^a^
	−	3 (16)	16 (84)	

Shown are the number (*n*) and percentage (%) of patients with hypomagnesemia by age group and *HNF1B* mutation status. The Fisher exact test compares the number of patients with hypomagnesemia across the mutation groups. Note that the frequency of hypomagnesemia increases with age and the difference between mutation groups becomes significant in the second half of childhood. Also note that individual patients may be represented in more than 1 age group, if their follow-up extended beyond this age group.

a Significant (*P* < 0.05) values.

Table 3 highlights the predictive values of a low plasma magnesium level in the different age quartiles and shows that absence of hypomagnesemia in the second half of childhood is highly predictive of not having a *HNF1B* mutation in children with renal tract malformations.

**Table 3:** Predictive values of hypomagnesemia for *HNF1B* mutation

Age (yr)	Positive predictive value	Negative predictive value
0–4.5	0.6	<0.5
4.5–9.0	0.5	<0.5
9.0–13.5	0.7	0.9
13.5–18.0	0.7	0.9

Shown are the positive and negative predictive values for hypomagnesemia and *HNF1B* mutation. Note, absence of hypomagnesemia in the second half of childhood is highly predictive of not having an *HNF1B* mutation in patients with renal tract malformations.

### Potassium, Chloride, and Bicarbonate

*HNF1B* mutations were associated with lower plasma potassium concentrations, but this was significant (*P* < 0.05) only in the oldest age group.

Similarly, plasma chloride concentrations trended lower with increasing age in mut+ patients, whereas bicarbonate concentrations increased with age in the mut+ group (Figure 2). However, the difference between the cohorts was not statistically significant and values outside the reference range were rare in both cohorts.

The cohorts were comparable with respect to plasma sodium and phosphate concentrations across the length of follow-up (data not shown).

### Urine Calcium/Creatinine Ratios

Values were available for only 22 patients and thus were too few for meaningful comparison across age and mutation status (Table 1).

### HNF1B Score

A total of 92 patients from this study had their HNF1B score calculated in the previous UK study.^8^ The median scores after adjustment for the latest available magnesium concentration was only slightly higher in patients older than 9 years compared with the younger patients, but the percentage of patients with a score ≥8, which had previously been suggested as a discriminator between mut+ and mut− patients, increased to more than 90% in the older age group (Table 4).^7^

**Table 4:** HNF1B score according to age

Age	<9 yr		>9 yr	
HNF1B mutation	+	−	+	−
Median score (*n*)	11 (13)	8 (37)	12 (13)	7.5 (28)
Score ≥8, %	77	51	92	50

Shown are the median HNF1B scores, as calculated previously,^8^ but adjusted for the latest available plasma magnesium concentration. Median scores are higher in the mut+ group, yet similar across the age groups. Note that the percentage of patients with a score ≥8 increases in the mut+ group with age, consistent with better discrimination between mut+ and mut− when using the suggested score cutoff of 8. For more details see text.

## Discussion

Our study provides important insights into the nature of the tubular dysfunction associated with *HNF1B* mutations and informs selection of pediatric patients for mutation analysis.

Most important, we show that hypomagnesemia develops with increasing age. Although there was a significant difference in median magnesium concentrations between mut+ and mut− patients across all age groups, overt hypomagnesemia was not apparent until age group 9.0 to 13.5 years. Moreover, the median age at which hypomagnesemia was first noted was 10.0 years (range 1.0–17.4 years). Thus, the absence of hypomagnesemia in younger children should not be used as an argument against testing for *HNF1B*, as the negative predictive value is low.

Next, we show that *HNF1B* mutations are not only associated with hypomagnesemia, but with a trend for a more complex pattern of electrolyte abnormalities comparable to Gitelman syndrome. In some patients, this can be quite dramatic, as in the boy reported previously, who presented marked electrolyte abnormalities with the typical pattern of Gitelman syndrome (K: 3.2, Cl: 97, and bicarbonate: 33 mmol/l) and consequently received this as his clinical diagnosis, yet on genetic testing was found to have an *HNF1B* mutation.^13^

Interestingly, as with magnesium, these Gitelman-like electrolyte abnormalities become apparent only with increasing age, so that the difference in potassium concentration between mut+ and mut− patients in our cohort became significant only after age 13.5 years. This may explain why in our initial review of children with *HNF1B* mutations, hypokalemia was not noted as a specific feature, whereas in a review of adult patients, approximately half were noted to have hypokalemia with renal potassium wasting, even despite worsening eGFR.^6^, ^12^ Hypomagnesemia may contribute to hypokalemia, as lack of intracellular magnesium unblocks the secretory potassium channel KCNJ1.^18^ However, in familial hypomagnesemia with secondary hypocalcemia due to mutations in *TRPM6*, hypokalemia has not been reported, arguing against a substantial contribution of hypomagnesemia to decreased potassium levels.^19^, ^20^

Our study has several limitations, including a relatively small sample size and the retrospective design with inconsistent availability of the various laboratory values. Individual patients may be represented in more than one age group, if data were available. Although this may bias the results toward those patients with multiple measurements, it also allows the tracking of the development of electrolyte abnormalities in individual patients, as shown for magnesium (Figure 3).

Differences in plasma chloride and bicarbonate concentrations and urine calcium were not significant in our study, and this likely reflects the small number of patients with available measurements (Table 1). Nevertheless, the trend for hypochloremia and alkalosis with increasing age is apparent in our data (Figure 2). Clinical symptoms potentially associated with the electrolyte abnormalities were not captured in this review. Autosomal dominant tubulointerstitial kidney disease–HNF1B is rare and larger cohort studies, ideally based on national or international registries, will be needed to overcome these limitations.

The DCT is the key nephron segment for magnesium regulation.^21^ HNF1B has been shown to regulate expression of *FXYD2*, which in turn regulates the activity of the basolateral Na^+^-K^+^-ATPase, the overall “engine” for all transport activity in this segment.^6^, ^10^ Thus, the biochemical phenotype is expected to be similar to that associated with mutations in FXYD2. Patients with mutations in this gene are exceedingly rare and only 1 mutation has so far been described. Initially, FXYD2 disease was described as a cause of isolated hypomagnesemia, yet subsequent data on newly discovered patients also show a trend to a Gitelman-like tubulopathy.^22^, ^23^ Interestingly, in a recent report of mutations in *ATP1A1*, encoding the alpha subunit of the Na^+^-K^+^-ATPase, hypomagnesemia was the predominant electrolyte abnormality, with hypokalemic alkalosis much less noticable.^24^ These data suggest that impaired activity of the Na^+^-K^+^-ATPase in DCT appears to primarily affect magnesium reabsorption. This also fits with the observation that impaired energy provision from mitochondria can also predominantly affect plasma magnesium levels.^25^

The finding of a slow evolution of the electrolyte abnormalities throughout childhood fits with clinical observations in other disorders of the DCT and raises interesting questions about the development of the role of this nephron segment. The archetypical disorder of impaired salt reabsorption in DCT is Gitelman syndrome and affected patients typically present during school age or even adulthood.^26^, ^27^, ^28^ A similar slow development of electrolyte abnormalities has been reported in a family with EAST/SeSAME syndrome.^29^ Gordon syndrome, the mirror image of Gitelman syndrome, also typically manifests later in life.^30^ Perhaps even more interesting is the clinical observation that patients with mutations in *CLCNKB* (Bartter syndrome type 3) often initially present with classical Bartter syndrome, but later in childhood may revert to a Gitelman-like phenotype.^31^ This chloride channel is expressed both in the thick ascending limb of Henle and in DCT, and the phenotypic switch to DCT-typical electrolyte abnormalities may represent the developing and increasing importance of salt reabsorption in this segment during childhood.^11^ Our clinical observations raise the question of whether HNF1B may actually be a transcriptional driver of this developmental change in apparent DCT activity. Yet, although there are several studies demonstrating the critical importance of HNF1B for kidney and especially also for tubular development, there are no data yet available to investigate the role of HNF1B in postnatal tubular maintenance and transport activity.^32^, ^33^, ^34^, ^35^, ^36^

## Conclusion

Our analysis of clinical data shows that the renal tubular dysfunction associated with mutations in *HNF1B* extends beyond isolated renal magnesium loss toward a Gitelman-like phenotype. Importantly, the electrolyte abnormalities associated with this tubulopathy develop during childhood and become most apparent in adolescence. The absence of these abnormalities in younger children with other suggestive findings thus does not argue against a potential underlying *HNF1B* mutation.

## Disclosure

All the authors declared no competing interests.
